# SampleExplorer: using language models to discover relevant transcriptome data

**DOI:** 10.1093/bioinformatics/btae759

**Published:** 2024-12-30

**Authors:** Wee Loong Chin, Timo Lassmann

**Affiliations:** National Centre for Asbestos Related Diseases, QEII Medical Centre, Nedlands, WA 6009, Australia; Department of Medical Oncology, Sir Charles Gairdner Hospital, Hospital Ave, Nedlands, WA 6009, Australia; The Kids Research Institute Australia, North Entrance, Perth Children's Hospital, 15 Hospital Ave, Nedlands, WA 6009, Australia; The Kids Research Institute Australia, North Entrance, Perth Children's Hospital, 15 Hospital Ave, Nedlands, WA 6009, Australia

## Abstract

**Motivation:**

Over the last two decades, transcriptomics has become a standard technique in biomedical research. We now have large databases of RNA-seq data, accompanied by valuable metadata detailing scientific objectives and the experimental procedures used. The metadata is crucial in understanding and replicating published studies, but so far has been underutilized in helping researchers to discover existing datasets.

**Results:**

We present SampleExplorer, a tool allowing researchers to search for relevant data using both text and gene set queries. SampleExplorer embeds sample metadata and uses a transformer-based language model to retrieve similar datasets. Extensive benchmarking (see Supplementary Materials and Methods) using the ARCHS4 database demonstrates that SampleExplorer provides an effective approach for retrieving biologically relevant samples from large-scale transcriptomicdata. This tool provides an efficient approach for discovering relevant gene expression datasets in large public repositories. It improves sample and dataset identification across diverse experimental contexts, helping researchers leverage existing transcriptomic data for potential replication or verification studies.

**Availability and implementation**: SampleExplorer is available as a Python package compatible with versions 3.9 to 3.11, available for installation via the Python Package Index (PyPI). The codebase and documentation are accessible at https://github.com/wlchin/SampleExplorer. Supplementary data (Supplementary Materials and Methods) provides detailed methodological information, including an algorithmic description of the retrieval process and data preparation steps.

## 1 Introduction

Functional genomics data has been accumulating at an unprecedented rate over the past decade, driven by advances in high-throughput sequencing technologies. However, our ability to analyze and interpret this data has not kept pace with its generation, resulting in a substantial fraction of available data remaining under-explored. In particular, transcriptome data generated in the context of one research study has the potential to be useful in another. Large volumes of transcriptome data are stored in large repositories such as Gene Expression Omnibus (GEO) (Clough and Barrett 2016), the European Bioinformatics Institute (EBI) (Emmert *et al.* 1994) Expression Atlas, and the ARCHS4 (Lachmann *et al.* 2018) database. ARCHS4 streamlines RNA-seq data analysis by providing uniformly pre-processed data alongside text-based metadata. The provided data enables researchers to bypass the time-consuming task of RNA-seq pre-processing and directly use count data for re-analysis. The accompanying metadata provides valuable experimental context, including extraction protocol and experimental conditions. These details are crucial for interpreting any results derived from the data.

Relevance or relatedness between experiments can be defined through two main lenses: transcriptional similarity and natural language similarity [also referred to as semantic similarity (Harispe *et al.* 2017)]. For this study, transcriptional similarity refers to shared gene expression patterns across samples from different experiments, while semantic similarity focuses on conceptual or thematic connections in experimental descriptions. An experiment can be considered relevant if it demonstrates either or both types of similarity to the experiment of interest. Importantly, these two forms of similarity do not always align; some experiments may be semantically related but produce different transcriptional outcomes, while others might yield similar transcriptional results despite differences in their descriptions. The most appropriate measure of relevance depends on the specific biological question being investigated, with both approaches offering valuable insights for functional genomics research.

Prior work focused on developing retrieval strategies that use transcriptome data to quantify transcriptional similarity, using metrics such as Euclidean distance and Pearson’s correlation coefficient (Glazko and Mushegian 2010, Miller and Bishop 2021, Hou *et al.* 2022). Other approaches have incorporated techniques like principal component analysis (PCA) (Zhang *et al.* 2024) or Kullback–Leibler divergence (Zhong *et al.* 2020) to identify similar samples. Recent online tools like Enrichr have made it easier to identify relevant samples, using gene set collections derived via automatic gene set discovery to retrieve samples from studies with similar transcriptional patterns. However, much less attention has been given to developing retrieval strategies that utilize metadata, which, although valuable, poses greater challenges due to its textual nature.

We hypothesize that combining transcriptome similarity and natural language search methods would help researchers identify relevant studies more effectively. To address this, we present SampleExplorer, a novel tool that incorporates two key contributions. First, it uses transformer-based language models (Naveed *et al.* 2023) (LMs) to process natural language queries and to augment search strategies by representing textual data more efficiently (Zakka *et al.* 2024, Gao *et al.* 2024, Jeong *et al.* 2024). Second, SampleExplorer combines these LM embeddings with transcriptome-based retrieval to enhance overall search effectiveness.

## 2 Materials and methods

*Data preparation*: To create embeddings of both transcriptome data and experimental metadata, we used the ARCHS4 Hierarchical Data Format version 5 (HDF5) data file accessed via the archs4py package (v0.2.19). We downloaded human RNA sequencing (RNA-seq) studies in the ARCHS4 (Lachmann *et al.* 2018) database (v2). This selection resulted in 22 207 well-documented bulk RNA-seq and single-cell RNA-seq studies spanning 722 425 samples.

*Generating the (text-based) metadata embeddings*: To create embeddings of experimental metadata, we acquired metadata directly from the ARCHS4 files. We supplemented this information by querying the National Center for Biotechnology (NCBI) Gene Expression Omnibus (GEO)1 database for titles, summaries, and overall design. After testing four LMs (Supplementary Tables S1 and S2, Supplementary Materials and Methods), we chose the all-mini-LM-v2 model to embed this textual metadata, creating a metadata database of 22 207 entries. Each metadata entry is represented by a 384D vector.

*Generating transcriptome embeddings*: To create low-dimensional embeddings of transcriptome data, we first aggregated condition-specific count data over experimental conditions in each study to generate average counts per gene. This resulted in a transcriptome database of 287 553 entries, with each reference transcriptome consisting of 67 186 genes. To reduce dimensionality while preserving inter-sample distances, we applied a Johnson–Lindenstrauss (Li *et al.* 2006, Lachmann *et al.* 2018) transform, creating dense embedding vectors containing 1000 elements each.

**Figure 1.**
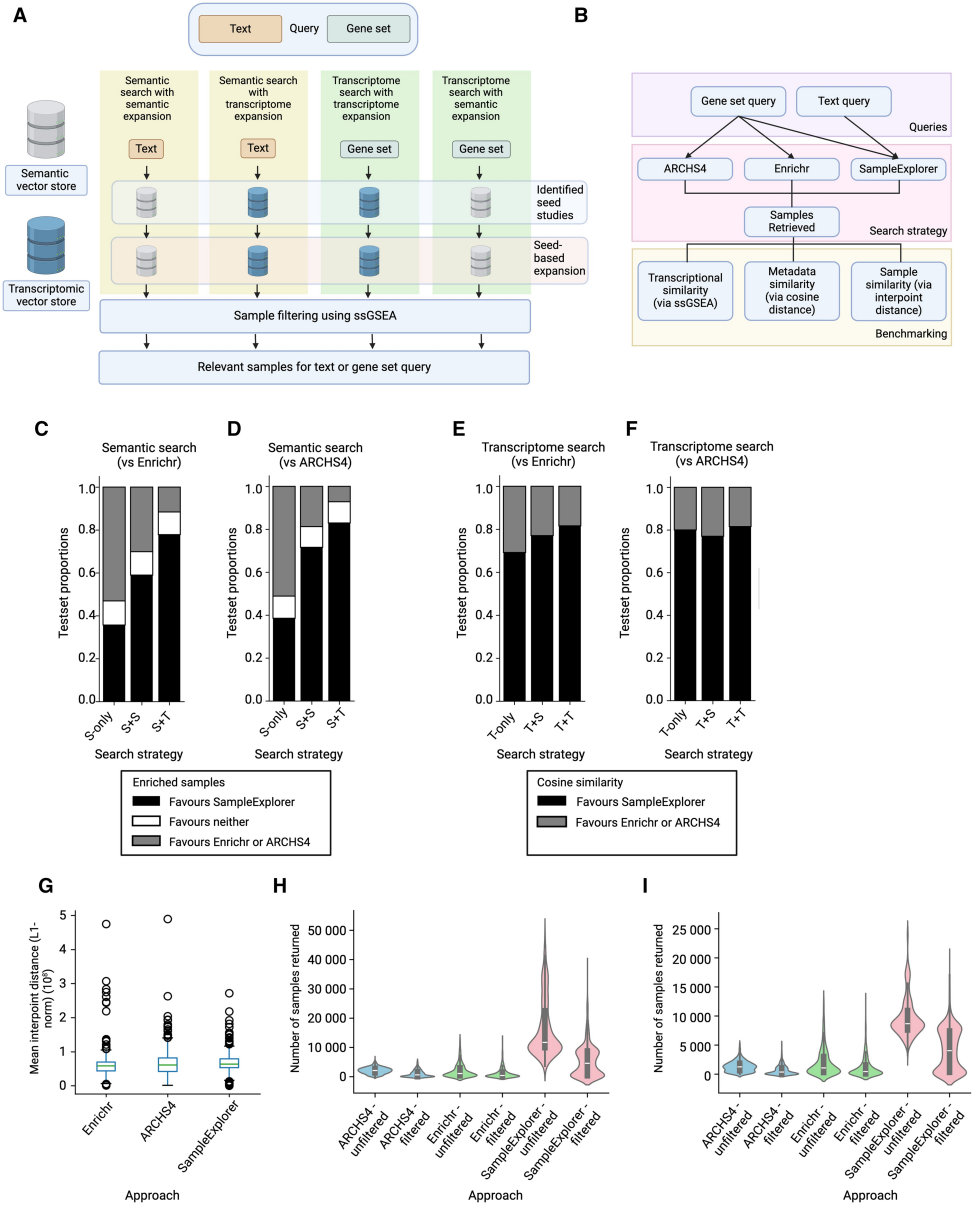
Workflow, benchmarking, and performance comparisons for SampleExplorer against other sample retrieval strategies. (A) Workflow diagram for SampleExplorer retrieval strategies. (B) Benchmarking strategy and metrics used to compare SampleExplorer to the ARCHS4 and Enrichr application programming interface (API). (C–F) Performance comparisons of SampleExplorer against reference APIs: SampleExplorer versus Enrichr using natural language search (C) and transcriptome search (E); SampleExplorer versus ARCHS4 using natural language search (D) and transcriptome search (F). In all comparisons, performance is expressed as a proportion of the test set where SampleExplorer outperformed or underperformed compared to the alternative method. Search strategies: S-only—natural language search; T-only—transcriptome search only; S + S, natural language search and expansion using transcriptome search; S + T—natural language search and expansion using transcriptome search. (G) Comparison of average interpoint distance between samples returned via three retrieval strategies. (H) Number of samples returned by each retrieval strategy, before and after filtering using ssGSEA. (I) Number of samples returned by each retrieval strategy, before and after filtering using ssGSEA, with single-cell samples excluded. For filtering, only samples statistically enriched for the query gene set (FDR < 0.05) are retained. Created in https://BioRender.com.

*Retrieval steps*: Key components of the workflow are illustrated in Fig. 1A. Our framework implements multiple retrieval strategies adapting to different search needs, with a core two-stage process that combines natural language search of metadata and transcriptome-based search.

1. *Natural language search*: In the first step, we search for candidate studies (experiments) using a textual query. This natural language search utilizes embedding lookup to find similar studies in the metadata embedding matrix. The textual query is converted into a vector representation using a LM, and the N-closest studies are returned based on cosine similarity as the distance metric.
2. *Transcriptome-based search*: In the second step, we expand the list of candidate studies through transcriptomic similarity. This process involves comparing the gene expression profiles of the initially retrieved studies with other studies in the transcriptome database. Again, we use embedding lookup and cosine similarity to identify the N-closest studies based on their transcriptomic profiles. This step helps to identify studies with similar molecular patterns, even if their metadata descriptions differ.

*Optional gene set enrichment analysis*: SampleExplorer allows users to optionally perform gene set enrichment analysis on samples from identified studies using their own gene set. This feature enables researchers to further refine their results by identifying studies where specific gene sets of interest are significantly enriched, providing an additional layer of biological relevance to the search results.

*Comparative analysis and benchmarking*: We evaluated SampleExplorer against two established strategies: gene set queries via the ARCHS4 Application Programming Interface (API), which uses Jordan–Lindenstrauss (Lachmann *et al.* 2018) transform for low-dimensional embedding projection, and the Enrichr API (Kuleshov *et al.* 2016), which uses statistical methods to compare query sets with predefined gene set libraries. Enrichr offers access to automatically generated gene sets from GEO studies, many of which are also included in the ARCHS4 database. To ensure a meaningful and consistent comparison, we used the ‘RNAseq_Automatic_GEO_Signatures_Human_Up’ library in Enrichr. This choice was made because it contains gene sets derived from the same GEO studies that are present in ARCHS4, focusing on upregulated genes in relevant biological contexts, allowing for a more direct and relevant comparison between SampleExplorer and existing tools. We assessed four retrieval sequences within SampleExplorer to identify the optimal approach: (i) natural language search followed by expansion using transcriptome similarity, (ii) transcriptome search followed by expansion using natural language similarity, (iii) natural language search followed by natural language similarity, and (iv) transcriptome search followed by expansion using transcriptome similarity.

*Test sets*: For our test set, we used the Molecular Signatures Database (MSigDB) repository (Liberzon *et al.* 2015), containing over 50 000 curated gene sets. We focused on 1000 gene sets from the C2 gene set collection of the MSigDB database, encompassing a diverse range of experiments across various biomedical fields. Each gene set in the MSigDB has an associated text description which describes the experimental condition used to derive the gene set.

*Performance metrics*: We evaluated SampleExplorer’s performance using four metrics and compared it with the ARCHS4 API and Enrichr API. The evaluation process encompassed both single-cell and bulk RNA-seq studies, with separate assessments conducted to account for the potentially higher sample numbers in single-cell studies.

1. *Relevance of retrieved samples*: To determine if SampleExplorer retrieved samples with transcriptome profiles relevant to the query, we computed the number of enriched samples for each gene set using single-sample Gene Set Enrichment Analysis (ssGSEA) (Supplementary Materials and Methods, Supplementary Fig. S1) from the decouplr-py package (version 1.6.1).
2. *Relevance of study metadata*: To assess if SampleExplorer retrieved samples in studies with metadata related to the user-supplied query, we measured average cosine similarity between query text and the textual metadata in candidate studies (Supplementary Materials and Methods, Supplementary Fig. S2).
3. *Interpoint distance analysis*: We quantified retrieval performance by measuring mean interpoint distances across both transcriptome and semantic spaces (Supplementary Materials and Methods, Supplementary Fig. S3). This analysis allowed us to evaluate whether retrieved samples showed similar patterns in both transcriptional and experimental contexts. For transcriptome-based retrieval, we calculated mean L1-normed distances between sample pairs in Johnson–Lindenstrauss embedding space and evaluated their relationships in semantic space. Conversely, for semantically retrieved samples, we computed mean pairwise distances between all-Mini-LM-v2 embedding vectors derived from experimental metadata and assessed their relationships in transcriptome space. Statistical significance between mean interpoint distances of retrieval strategies was assessed using Wilcoxon and Fisher’s exact tests.
4. *Sample set similarity from various retrieval strategies*: To measure the similarity of sample sets retrieved from SampleExplorer, the ARCHS4 API, and the Enrichr API, we used normalized Jaccard distance (Vorontsov *et al.* 2013), with higher distances indicating greater dissimilarity (Supplementary Table S3, Supplementary Materials and Methods).

*Metric selection: independence and complementarity*: While our evaluation metrics provide useful insights into retrieval performance, it is important to acknowledge their limitations and potential biases:

1. ssGSEA-based enrichment analysis may favour methods that explicitly use transcriptional similarity in their retrieval process, as both SampleExplorer and the ARCHS4 API leverage gene expression patterns. To address this potential bias, we also evaluated performance using metrics not used as part of the retrieval mechanism, specifically the L1-normed mean point-to-point distance between samples.
2. Cosine similarity in metadata space could potentially favour SampleExplorer’s natural language search component since it uses similar embedding techniques. This metric primarily serves to verify that retrieved samples maintain semantic relevance to the query, rather than as a direct performance comparison.

Given these limitations, interpoint distances evaluated using transcriptome-based retrieval and semantic retrieval provide complementary evaluation metrics that are not used in the retrieval process.

*Hyperparameter tuning*: The impact of sample numbers retrieved during the search and expansion steps was evaluated using a grid search. This utilized the first two metrics, with final hyperparameter values chosen based on manual inspection of the hyperparameter grids (Supplementary Materials and Methods, Supplementary Fig. S4). This process was conducted on a separate set of 100 gene sets from the MSigDB C2 gene set collection, distinct from our test set. The chosen hyperparameter values are provided in Supplementary Table S4 (Supplementary Materials and Methods).

## 3 Results

### 3.1 Evaluating retrieval sequence using SampleExplorer

We explored the best way to retrieve samples with SampleExplorer. We compared four methods of retrieval (Fig. 1A), each with different sequences for searching through the transcriptome database or metadata database. To determine which method was more effective, we analyzed two metrics: the number of enriched samples by ssGSEA (Supplementary Materials and Methods, Supplementary Fig. S1) and the average similarity to our queries (Supplementary Materials and Methods, Supplementary Fig. S2). For each item in the test set, if SampleExplorer performed better than another method, we considered it in favour of SampleExplorer. We then calculated the proportion of queries where SampleExplorer outperformed or underperformed compared to the alternative method. We found SampleExplorer performed better than other retrieval strategies when both the metadata and transcriptome databases were both used for retrieval (Fig. 1B–E). Among the different search sequences, natural language search with transcriptome expansion demonstrated superior performance compared to the ARCHS4 API or the Enrichr API (Fig. 1B–E), with up to 80% of the queries favouring SampleExplorer in our comparative analysis. As a result of these experiments, we established the default retrieval strategy in SampleExplorer to be textual search against the metadata database, followed by expansion of the candidate study list using the transcriptome database.

### 3.2 Assessing sample characteristics from different retrieval strategies

We analyzed the differences and similarities of the samples retrieved using SampleExplorer, the Enrichr API or the ARCHS4 API using our previously described 1000-item test set. Using mean interpoint distance (Fig. 1G), there were no significant differences in sample distribution among these three retrieval strategies. We compared the average interpoint distances between samples returned by the three retrieval strategies and found no significant differences in their distributions (Fig. 1G). Detailed analysis of these distances (see Supplementary Materials and Methods) revealed that SampleExplorer’s search yielded samples with greater transcriptional diversity and more closely related metadata compared to existing approaches. Evaluating sample overlap using average Jaccard distance, there was little overlap between sample sets retrieved by the three methods (Supplementary Table S3, Supplementary Materials and Methods). These observations indicate that each strategy prioritized retrieval of distinct sets of similar samples within the ARCHS4 database. When we compared the total number of retrieved samples per query to the number of enriched (relevant) samples using ssGSEA, SampleExplorer retrieved more samples per query, resulting in a higher number of enriched samples than the Enrichr API or the ARCHS4 API (Fig. 1H). We continued to observe this even when we excluded single-cell data from the analysis (Fig. 1I). Taken together, we interpret these results to indicate that SampleExplorer, the Enrichr API, and the ARCHS4 API use complementary approaches that prioritize retrieval of distinct subsets of samples from the ARCHS4 database, with SampleExplorer demonstrating an advantage in retrieving a higher number of samples relevant to a given query.

### 3.3 Discussion

SampleExplorer leverages transformer-based LMs to create searchable, low-dimensional representations of experimental metadata, enhancing retrieval of relevant studies by augmenting gene set queries with textual information. Building on data curation initiatives like ARCHS4 and publicly available scientific data, our tool aims to facilitate meta-analyses and cross-validation of findings across multiple datasets.

The performance of SampleExplorer across multiple metrics should be considered within the context and limitations of our evaluation framework. While ssGSEA and cosine similarity measurements share some commonality with our retrieval strategy, the consistent advantages observed in mean L1-normed distance (an independent metric) suggest genuine improvements rather than evaluation artefacts. Furthermore, high Jaccard distances (>0.99) between methods indicate that SampleExplorer, the ARCHS4 API, and the Enrichr API each identify distinct sets of relevant samples from the ARCHS4 database. This minimal overlap, combined with the observation that each method successfully retrieves samples showing enrichment for query gene sets, suggests multiple valid approaches exist for sample identification.

By improving the identification of relevant studies, SampleExplorer contributes to the derivation of more robust and widely applicable scientific conclusions in functional genomics research.

## Supplementary Material

btae759_Supplementary_Data

## Data Availability

The software implementation and analysis code associated with this article are available through GitHub (https://github.com/wlchin/SampleExplorer) and have been archived at Zenodo (10.5281/zenodo.14233740). The source data were obtained from the ARCHS4 database at (https://maayanlab.cloud/archs4/).
