# Supplementary material for: SampleExplorer: using language models to discover relevant transcriptome data
Source: Bioinformatics. 2024 Dec 30;41(1):btae759. doi: 10.1093/bioinformatics/btae759 (PMC11751629; doi:10.1093/bioinformatics/btae759)
Supplement: btae759_Supplementary_Data [file btae759_supplementary_data.zip › d1baf_Supplementary data.pdf]

Supplementary data (Materials and Methods)

Introduction 2
Data preparation 2
Data sources and software 2
Generating the (text-based) metadata embeddings. 3
Generating transcriptome embeddings. 4
Implementation 4
Natural Language (Semantic) Search Implementation 5
Transcriptome Search Implementation 5
Combining Semantic and Transcriptome Search 5
Semantic + Semantic Search 5
Semantic + Transcriptome Search (Default) 5
Transcriptome + Transcriptome Search 6
Transcriptome + Semantic Search 6
Software Implementation 6
Benchmarking and Evaluation 6
Embedding Models and Evaluation 6
Database benchmarks 7
ARCHS4 database 7
Enrichr Web API 7
Test Dataset 8
MSigDB C2 signatures 8
Example C2 signature 9
Evaluation Metrics 9
Number of enriched samples 9
Average semantic similarity 10
Transcriptional similarity 10
Sample set overlap 10
Extended Analysis of Sample Distance Metrics 10
Experiments 11
Hyperparameter Optimisation for SampleExplorer 11
Retrieval Strategy Evaluation 11
Extended analysis of interpoint distance metrics 12
Single-Cell Data Analysis 13
Sample Characteristics Analysis 13
Figures and tables 14

Abbreviations 22
References 22

Introduction

We introduce SampleExplorer, a novel tool for semantic (natural language) and transcriptome-based retrieval of studies and samples from the ARCHS4 (Lachmann *et al.*, 2018) database. SampleExplorer combines natural language processing with transcriptome information to identify studies relevant to a user's query, both conceptually and in terms of gene expression patterns.

The primary data source for SampleExplorer is the ARCHS4 database, which contains uniformly processed RNA sequencing data from human studies available in the Gene Expression Omnibus (GEO) (Clough and Barrett, 2016).

Here we outline the workflow for data pre-processing and embedding generation. It describes the natural language search, the transcriptome search, and our benchmarking strategy. We believe that SampleExplorer will serve as a valuable resource for researchers and clinicians seeking to identify relevant studies within the ARCHS4 database.

Data preparation

Data sources and software

To generate embeddings for both transcriptome data and experimental metadata, we utilised publicly available pre-processed data accessed through the archs4py package (version 0.2.19). We obtained human RNA sequencing (RNA-seq) studies from the ARCHS4 (Lachmann *et al.*, 2018) website, specifically using version 2 of the human RNA-seq database in Hierarchical Data Format version 5 (HDF5) format. This comprehensive HDF5 file contains 22,207 well-documented Gene Expression Omnibus (GEO) studies, encompassing both bulk RNA-seq and single-cell RNA-seq data, and indexes 722,425 samples.

The HDF5 file is structured into two primary groups: “data” and “meta”. The data section contains a two-dimensional array of shape (67186, 722,425) and is stored as 32-bit unsigned integers (uint32). This dataset represents a large matrix of gene expression data, with rows corresponding to genes and columns to samples. The “meta” section contains sample metadata. The metadata schema for each sample in the ARCHS4 database comprises 30 distinct fields, as detailed in Supplementary Table 1.

Generating the (text-based) metadata embeddings.

To create embeddings of experimental metadata, we implemented a multi-step process that combines data from both the ARCHS4 and GEO databases. We first extracted the GEO series ID (GSEID) information from the ARCHS4 files. For each series ID, we then used the GEOparse library (v2.0.4) to collect additional metadata from the GEO database, specifically retrieving the title, summary, and overall design for each GSEID and concatenating these text-based descriptions into a single metadata entry to be processed by the embedding model. This approach allowed us to integrate the data available in ARCHS4 with the descriptive information from GEO.

We evaluated four different embedding models using the Retrieval-Augmented Generation Assessment (RAGAs) framework (Es *et al.*, 2023). RAGAs is designed to assess the performance of RAG (Retrieval-Augmented Generation) pipelines, which enhance language models (LMs) by integrating retrieval and generation components. Key metrics for evaluation include context relevancy and context recall, which contribute to a comprehensive RAGAs score. The performance results for different embedding models are presented in Supplementary Table 2. As the RAGAs assessment showed no significant under- or over-performance among the models, we opted for the most accessible and general-use open-source option: the all-mini-LM-v2 embedding model.

The final output is a metadata database comprising 22,207 entries, each corresponding to every unique GSEID in the ARCHS4 database. Each metadata entry is represented by a 384-dimensional vector, forming the embedding matrix for experimental metadata.

Generating transcriptome embeddings.

To create low-dimensional embeddings of transcriptome data, we employed a multi-step process. First, we aggregated condition-specific count data over experimental conditions within each study to generate average counts per gene, using raw read counts as provided in the ARCHS4 HDF5 file. This aggregation process combines count data from all technical replicates for each unique condition within a study, identified by its GEO identifier. We calculated the mean count per gene across all replicates for each condition, using the "characteristics_ch1" column (see Supplementary Table 1) to group data by GSEID and characteristic. This approach resulted in a transcriptome database of 287,553 entries, with each reference transcriptome consisting of 67,186 genes.

To reduce the dimensionality of these reference transcriptomes while preserving inter-sample distances, we applied a Johnson-Lindenstrauss transform. This process created dense embedding vectors containing 1,000 elements each, resulting in a transcriptome database matrix of 287,553 x 1,000 entries.

Implementation

There are two types of searches in SampleExplorer, which are described below. Our retrieval strategy employs the concept of "seed studies", which serve as query vectors in embedding space for retrieving similar samples or studies based on cosine similarity. We define seed studies as initial reference points derived from the user's query or the first stage of our search process. These seed studies are crucial for guiding subsequent searches, whether in semantic or transcriptome space. In the context of semantic searches, seed studies are those that closely match the user's textual query in concept space. For transcriptome searches, seed studies are those with gene expression patterns most similar to the query gene set. The use of seed studies allows us to iteratively expand our search results, expanding from an initial set of closely related studies to a broader, yet still relevant, collection of relevant samples within the ARCHS4 database.

Natural Language (Semantic) Search Implementation

The SampleExplorer tool implements a semantic search component using natural language processing to match user queries with relevant studies. This process involves three main steps. First, we generate a 384-dimensional dense vector embedding for the pre-processed user query using the all-MiniLM-L6-v2 model from Sentence-Transformers (v3.0.1). Next, we compute the cosine similarity between this query embedding and pre-existing embeddings in our database, assigning a similarity score to each study. Finally, we rank the studies based on these similarity scores and retrieve the top N most similar studies, where N is a hyperparameter optimised during our evaluation process.

Transcriptome Search Implementation

SampleExplorer's transcriptome search component operates on a Johnson-Lindenstrauss (JL) transformed matrix of reference transcriptomes, which reduces dimensionality while approximately preserving pairwise distances between points. Using this transformed matrix, we identify studies with similar gene expression patterns in two ways based on the query mode. For gene set queries, we use single-sample Gene Set Enrichment Analysis (ssGSEA) to derive the top N transcriptomes enriched for the user's genes. For natural language (semantic) queries which yield ‘seed studies’ based on similarity in metadata embedding space, we then subsequently retrieve the N closest transcriptomes to these seeds based on cosine similarity in the JL-transformed embedding space, where N is a hyperparameter optimised during our evaluation process.

Combining Semantic and Transcriptome Search

Semantic + Semantic Search

We perform an initial semantic search using the user's query to identify conceptually related studies. These initial results then serve as seed studies for a secondary semantic search, expanding the pool of conceptually related studies.

Semantic + Transcriptome Search (Default)

We begin with a semantic search to identify conceptually relevant studies. The top-ranked studies from this search become seed studies, and we extract their associated

reference transcriptomes. We then conduct a transcriptome search using these seed transcriptomes to find studies with similar gene expression patterns.

Transcriptome + Transcriptome Search

This two-stage process starts with an initial transcriptome search using the query gene set. We apply single-sample Gene Set Enrichment Analysis (ssGSEA) to identify the top 1000 studies. The highest-ranking results from this ssGSEA analysis then serve as seed data for a secondary transcriptome search.

Transcriptome + Semantic Search

We begin with a transcriptome search using ssGSEA to identify studies with similar gene expression patterns to the query. We map these transcriptomes to their corresponding GSEIDs. These GSEIDs then serve as the basis for a semantic search on the metadata of the transcriptionally similar studies.

In all methods, the embedding vector (query) is used to retrieve elements in embedding space based on cosine similarity, allowing for efficient and relevant study retrieval.

Software Implementation

SampleExplorer is implemented as a Python package compatible with versions 3.9 to 3.11. It is available for installation through PyPI, with the codebase and documentation accessible at https://github.com/wlchin/SampleExplorer. SampleExplorer is also available via a containerised Streamlit (https://streamlit.io/) application. The application implements natural language search and transcriptome expansion, which we found in our benchmarks (described below) to perform best for retrieving relevant experimental samples.

Benchmarking and Evaluation

Embedding Models and Evaluation

To embed the textual metadata, we evaluated four large language models: the text-embedding-ada-002 model from OpenAI, open-source models trained on scientific textual data (e.g., SciBERT, ClinicalBERT), and the all-MiniLM-L6-v2 model (default

in Sentence-Transformers 2.5.1 package). The evaluation was conducted using the Ragas (v0.0.22) package, which generated a synthetic dataset of 100 question-answer pairs using GPT-3.5-turbo-1160. Performance was assessed using context precision and recall metrics. Our results showed no significant difference between the models in terms of performance. Given this, we retained the all-MiniLM-L6-v2 model for our pipeline due to its balance of performance and efficiency.

Database benchmarks

We evaluated SampleExplorer's performance against two established strategies for querying the ARCHS4 database, both accessible via web-based application programming interfaces (APIs).

ARCHS4 database

For gene signature queries, we utilise the ARCHS4 API, which employs the Johnson-Lindenstrauss (JL) transform (Lachmann *et al.*, 2018; Li *et al.*, 2006) to project user-provided gene sets onto a low-dimensional embedding matrix. This dimensionality reduction technique preserves approximate pairwise distances between points in the high-dimensional space, enabling efficient similarity computations. The API then returns a list of ARCHS4 samples similar to the query gene set.

It is important to note that this API functionality is limited to gene set queries only; text-based queries are not supported according to the available documentation.

Enrichr Web API

For our comparative analysis evaluating methods of identifying relevant studies and samples, we employed Enrichr (Kuleshov *et al.*, 2016), a web-based tool designed for gene set enrichment analysis that enables the identification of statistically overrepresented biological pathways, transcription factors, and functional categories in gene sets derived from genome-wide experiments. Enrichr identifies relevant studies through gene set overlap analysis using the Fisher's exact test (Kuleshov *et al.*, 2016). Among Enrichr's 232 gene set libraries, several libraries contain study-specific gene sets automatically derived from GEO experiments, enabling users to identify GEO studies which show enrichment for their query gene sets.

We queried Enrichr through its API using the gseapy (v1.1.3) Python package. For evaluating retrieved studies, we specifically employed Enrichr's "RNAseq_Automatic_GEO_Signatures_Human_Up" library (4,269 gene sets), which contains study-specific gene sets derived from human RNA-seq experiments in the Gene Expression Omnibus (GEO) database, focusing on upregulated genes. The Enrichr API returns enriched biological terms (gene sets indexed by source study), p-values, and adjusted p-values for multiple testing, identifying significantly enriched studies for each query. Since these studies are represented in both the Enrichr gene set library and the ARCHS4 database, we could directly compare the biological relevance of studies retrieved by each method.

Test Dataset

Our test dataset consisted of 1000 gene sets from the C2 gene set collection of the Molecular Signatures Database (MSigDB) (Liberzon *et al.*, 2011). These gene sets served as standardised queries to evaluate the ability of each method to retrieve biologically relevant samples using our performance metrics.

MSigDB C2 signatures

Our evaluation of SampleExplorer utilised a test dataset comprising 1000 gene sets randomly selected from the C2 gene set collection of the Molecular Signatures Database (MSigDB). Crucially, each gene set in the MSigDB has an associated text description detailing the experimental condition used to derive the gene set, allowing us to benchmark performance using both gene set and natural language retrieval.

This choice of test set was motivated by two key factors. First, the C2 collection's diversity, sourced from various pathway databases, publications, and expert knowledge, ensures a comprehensive evaluation across different molecular biology domains. Second, these gene sets, derived from experimental data and curated pathways, provide biologically relevant queries that reflect real-world scenarios.

This test dataset enables a thorough evaluation of SampleExplorer's ability to retrieve meaningful and physiologically relevant data across a spectrum of biological processes and experimental conditions.

Example C2 signature

To illustrate the nature of our test dataset, we can consider an example gene set from the C2 collection: BENITEZ_GBM_PROTEASOME_INHIBITION_RESPONSE. This gene set is derived from a study (Benitez *et al.*, 2021) on glioblastoma (GBM), the most common primary brain tumour in adults. The researchers conducted a high-throughput drug screen using patient-derived neurospheres cultured to retain their glioblastoma stem cell (GSC) phenotype. They discovered that GSCs were highly sensitive to proteasome inhibition, particularly in cells with PTEN loss and activation of the PI3K/mTOR pathway. The gene set specifically represents 102 proteins that accumulated after proteasome inhibition with carfilzomib in PTEN-knockout GBM spheres, compared to placebo-treated and PTEN wild-type groups. This proteomic signature was correlated with the RNA-level transcriptomic response to carfilzomib using Gene Set Enrichment Analysis (GSEA), with the leading edge of the enrichment result selected as the response signature. This example demonstrates the biological relevance and complexity of the gene sets in our test dataset, derived from actual experimental data (GSE163906) and representing specific biological processes and therapeutic responses.

Evaluation Metrics

We employed four primary evaluation metrics to provide a comprehensive assessment of SampleExplorer's performance:

Number of enriched samples

We used single-sample Gene Set Enrichment Analysis (ssGSEA) from the decouplr-py package (version 1.6.1) to calculate the number of statistically enriched samples for each gene set (Supplementary Figure 1). This metric helped determine if SampleExplorer retrieved samples with transcriptome profiles relevant to the query. Samples were deemed enriched with a gene set corresponding to an adjusted p-value of < 0.05. In our benchmarking workflows, a query strategy was deemed better if it produced more statistically enriched samples than another method.

Average semantic similarity

We measured the average semantic similarity between the query text and the textual metadata in candidate studies using cosine similarity. This metric assessed whether SampleExplorer retrieved samples from studies with metadata related to the user-supplied query (Supplementary Figure 2). In our benchmarking workflows, a query strategy was deemed better if it consistently produced higher average semantic similarity scores. This indicates that the retrieved samples are more closely related to the query in terms of their associated metadata, suggesting a stronger conceptual match between the query and the retrieved studies.

Transcriptional similarity

We calculated the L1-normed mean point-to-point distance (Moravec, 2015; Glazko and Mushegian, 2010) between samples returned by each search strategy. This metric measured the overall transcriptional similarity between retrieved samples for each item in our test set (Supplementary Figure 3). A lower L1-normed mean point-to-point distance indicates that the retrieved samples have more similar transcriptional profiles, suggesting that the strategy is more consistent in identifying samples with related gene expression patterns. Notably, this measure only evaluates relationships between retrieved results and does not directly assess relevance to the query.

Sample set overlap

We used normalised Jaccard distance to measure the similarity between sets of samples retrieved from SampleExplorer, the ARCHS4 API, and the Enrichr API. Higher distances indicated more dissimilarity between the retrieved sample sets (Supplementary Table 3).

Extended Analysis of Sample Distance Metrics

To further evaluate retrieval performance, we implemented a distance-based evaluation framework that complements our primary analysis metrics (ssGSEA and cosine similarity). This framework provides independent measures of result coherence in both transcriptomic and semantic spaces, offering additional insight into the effectiveness of different retrieval strategies.

Our primary metric for quantifying cluster cohesion was the L1-normed mean point-to-point distance between sample pairs, calculated in two distinct spaces. In transcriptome space, defined by Johnson-Lindenstrauss (JL) embedding vectors of transcriptional profiles (Supplementary Figure 3), this metric captures relationships between gene expression patterns. In semantic space, defined by all-Mini-LM-v2 embedding vectors derived from experimental metadata, it measures relationships between experimental descriptions (see also section on Extended Analysis of Interpoint Distance Metrics). Smaller interpoint distances in transcriptome space indicate higher similarity in gene expression profiles, while in semantic space they reflect greater consistency in experimental conditions. Conversely, larger distances indicate greater transcriptional diversity or more varied experimental conditions, respectively.

To avoid circularity in our evaluations, we tested the semantic similarity of samples retrieved by transcriptome-based search and, conversely, the transcriptional similarity of samples retrieved by semantic search. This evaluation strategy provided insights into how well each method maintained relevance across both biological and experimental domains.

Experiments

Hyperparameter Optimisation for SampleExplorer

Having selected the all-Mini-LM-v2 embedding model based on previous benchmarks (see section on Embedding Models and Evaluation), we conducted a grid search to evaluate the impact of the number of samples retrieved during search and expansion steps. We employed the first two metrics (number of enriched samples and average semantic similarity) for this hyperparameter optimisation process. The choice of final hyperparameter values (Supplementary Table 4) was based on manual inspection of the hyperparameter grids (Supplementary Figure 4) on a separate set of 100 gene sets from the MSigDB C2 gene set collection, distinct from our main test set.

Retrieval Strategy Evaluation

We explored distinct methods of retrieval to determine the most effective approach:

1. Semantic search with semantic expansion
2. Semantic search with transcriptome expansion
3. Transcriptome search with transcriptome expansion
4. Transcriptome search with transcriptome expansion

For each item in the test set, we compared SampleExplorer's performance using these methods against the ARCHS4 API and Enrichr API. If SampleExplorer performed better than another method, we considered it in favour of SampleExplorer. We then calculated the proportion of queries where SampleExplorer outperformed or underperformed compared to the alternative methods.

Extended analysis of interpoint distance metrics

Statistical comparison of retrieval methods using paired Wilcoxon tests revealed significant differences in performance across spaces (Supplementary Table 5). In semantic space (all-Mini-LM-v2 vectors), comparisons showed significant differences between ARCHS4 and SE (1-step) ($p = 6.70e-08$), as well as between Enrichr and both SE (1-step) ($p = 9.16e-47$) and SE (2-step) ($p = 2.69e-37$). In transcriptome space (JL vectors), both SE (1-step) and SE (2-step) showed significant differences compared to ARCHS4 ($p = 3.03e-13$ and $p = 1.89e-26$, respectively) and Enrichr ($p = 2.03e-02$ and $p = 3.56e-14$, respectively).

Using Fisher's exact test, we examined how mean interpoint distances related to established metrics (Supplementary Table 6). In transcriptome space, smaller interpoint distances correlated with lower ssGSEA enrichment scores, with strong statistical significance in Enrichr comparisons (SE (2-step): OR = 0.525, $p = 2.03e-04$; SE (1-step): OR = 0.279, $p = 4.34e-18$) and marginal significance in ARCHS4 comparisons (SE (2-step): OR = 0.756, $p = 8.29e-02$; SE (1-step): OR = 0.777, $p = 6.14e-02$). In semantic space, we found no significant relationships between interpoint distances and cosine similarity metrics.

Key findings from our analysis revealed that SE (1-step) and SE (2-step) semantic-based retrieval demonstrated larger JL vector distances, indicating greater transcriptional diversity among retrieved samples. Conversely, both SE approaches in all-Mini-LM-v2 vector space showed more closely related metadata compared to

Enrichr, while only SE (1-step) showed significantly more closely related metadata compared to ARCHS4 (with SE (2-step) showing no significant difference). We found that smaller interpoint distances showed lower ssGSEA enrichment scores in both ARCHS4 and Enrichr comparisons, while Mini-LM-v2 vector distances showed no relationship with cosine similarity scores.

Single-Cell Data Analysis

Recognising that single-cell studies potentially contain more samples per study than bulk RNA-seq studies, we conducted our performance assessments both with and without single-cell data included. We excluded single-cell data in our assessments using the "remove_sc" flag for samples retrieved using the archS4py package.

Sample Characteristics Analysis

To understand the differences and similarities of samples retrieved using SampleExplorer, the Enrichr API, and the ARCHS4 API, we conducted several analyses. We used L1-normed mean point-to-point distance (Glazko and Mushegian, 2010; Moravec, 2015) to assess sample distribution (Supplementary Figure 5) and average Jaccard distance to evaluate sample overlap (Supplementary Table 3). We also compared the total number of retrieved samples to the number of enriched (relevant) samples. These analyses were performed both including and excluding single-cell data to ensure robustness of our findings across different data types (Supplementary Figure 5).

Supplementary Figures

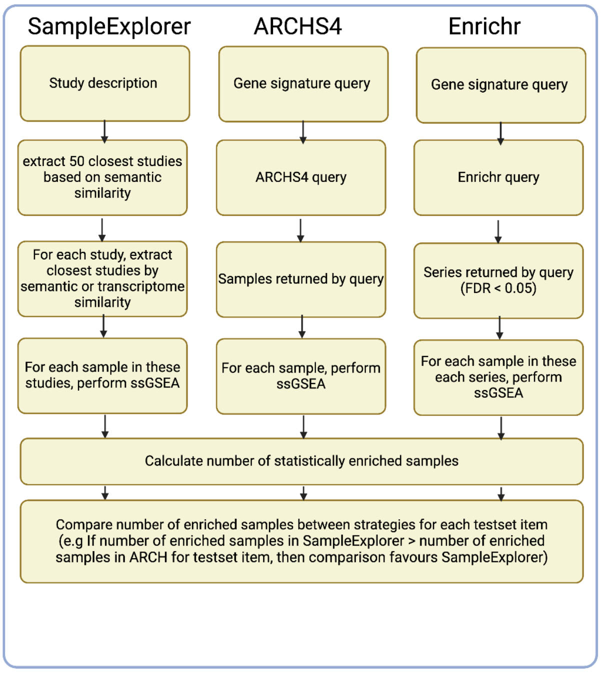

Supplementary Figure 1. Benchmarking SampleExplorer semantic search against Enrichr and ARCHS4 APIs: The performance of SampleExplorer's natural language search capabilities was evaluated against the Enrichr and ARCHS4 APIs in retrieving biologically relevant samples based on gene set enrichment. Samples returned from all three methods are evaluated using single-sample Gene Set Enrichment Analysis (ssGSEA). Samples with a corrected p-value of < 0.05 using ssGSEA are considered statistically enriched. This benchmark aims to evaluate whether SampleExplorer retrieves a larger number of samples with statistically significant gene set enrichment compared to Enrichr and ARCHS4, thereby assessing the effectiveness of SampleExplorer's approach in identifying biologically relevant samples.

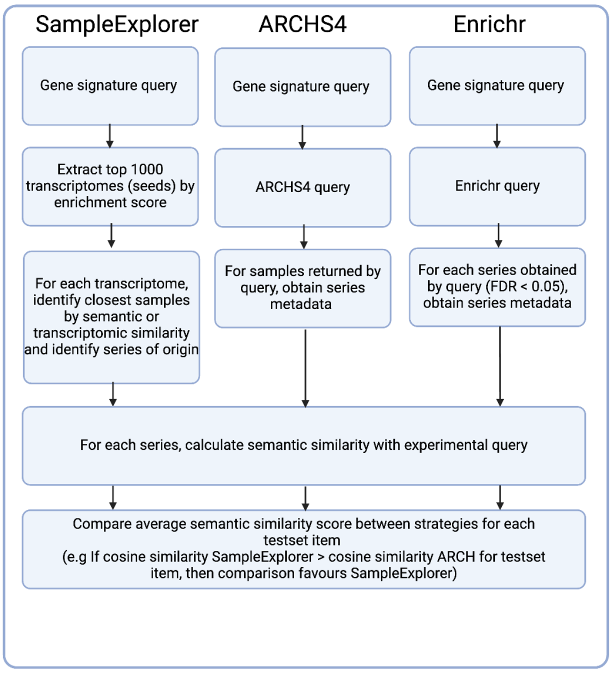

Supplementary Figure 2. Benchmarking SampleExplorer transcriptome search against the Enrichr and ARCHS4 API. Semantic (natural language) similarity, quantified using cosine distance, was used to measure the experimental relevance of results returned from each retrieval strategy. For all three methodologies, the samples returned from queries were mapped to their GEO experimental descriptions. The average semantic similarity between these experimental descriptions and the initial natural language query (the text description accompanying the gene set in the test set) was computed using cosine distance in embedding space. Higher average semantic similarity scores indicated better response quality, reflecting greater concordance between retrieved sample experimental descriptions and the original query.

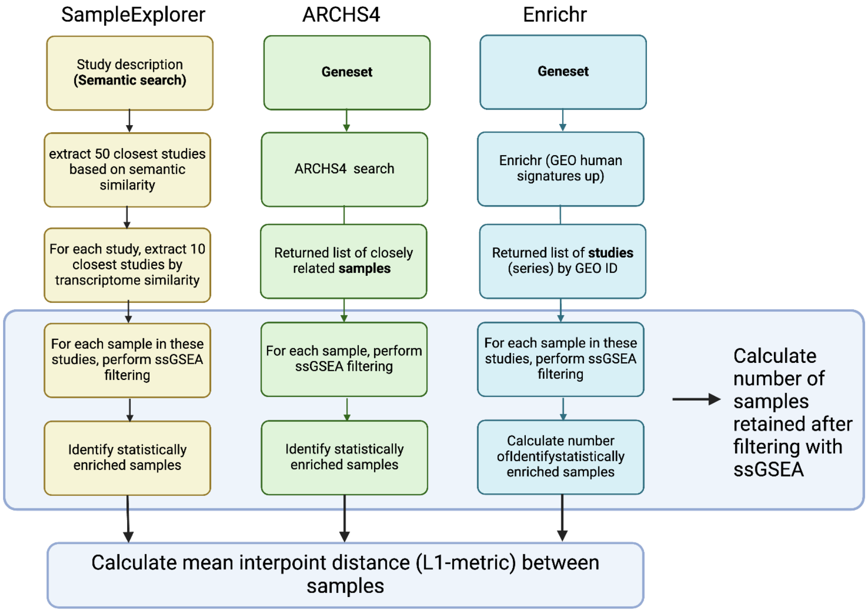

Supplementary Figure 3. Comparing sample diversity retrieved via different embedding strategies. This workflow evaluated the transcriptional diversity of samples returned by various query methods. Relevant samples were first obtained from each search strategy. Subsequently, sample diversity was quantified by calculating the mean point-to-point distance using the L1 metric for each method's returned sample transcriptomes. This metric serves as a proxy for sample diversity, with higher L1 distances indicating greater diversity and potentially broader representation of biological contexts related to the query.

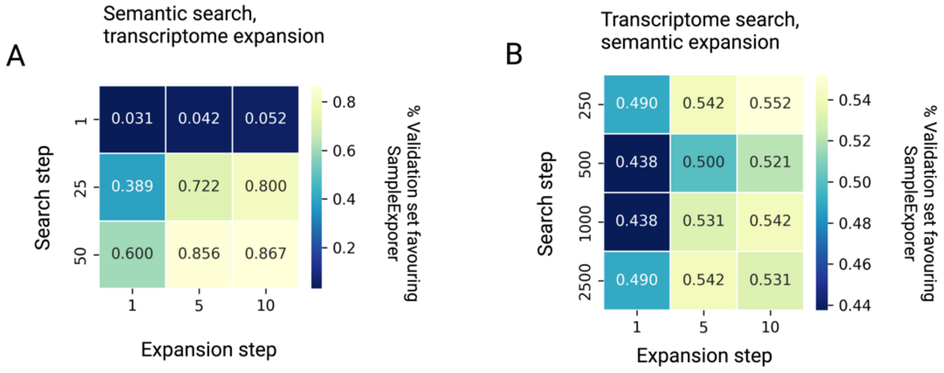

Supplementary Figure 4. Hyperparameter optimisation for SampleExplorer retrieval strategies. SampleExplorer's two-step retrieval approach allows for the specification of different values for the number of seed and expansion studies. A grid search was performed to find optimal default values for the benchmarking experiments performed in Supplementary Figures 1 and 2. SampleExplorer's semantic and transcriptome searches were compared against the ARCHS4 API. These experiments utilised a distinct validation set of 100 gene sets, separate from the 1000 gene sets in the Molecular Signatures Database (MSigDB) referenced in previous analyses.

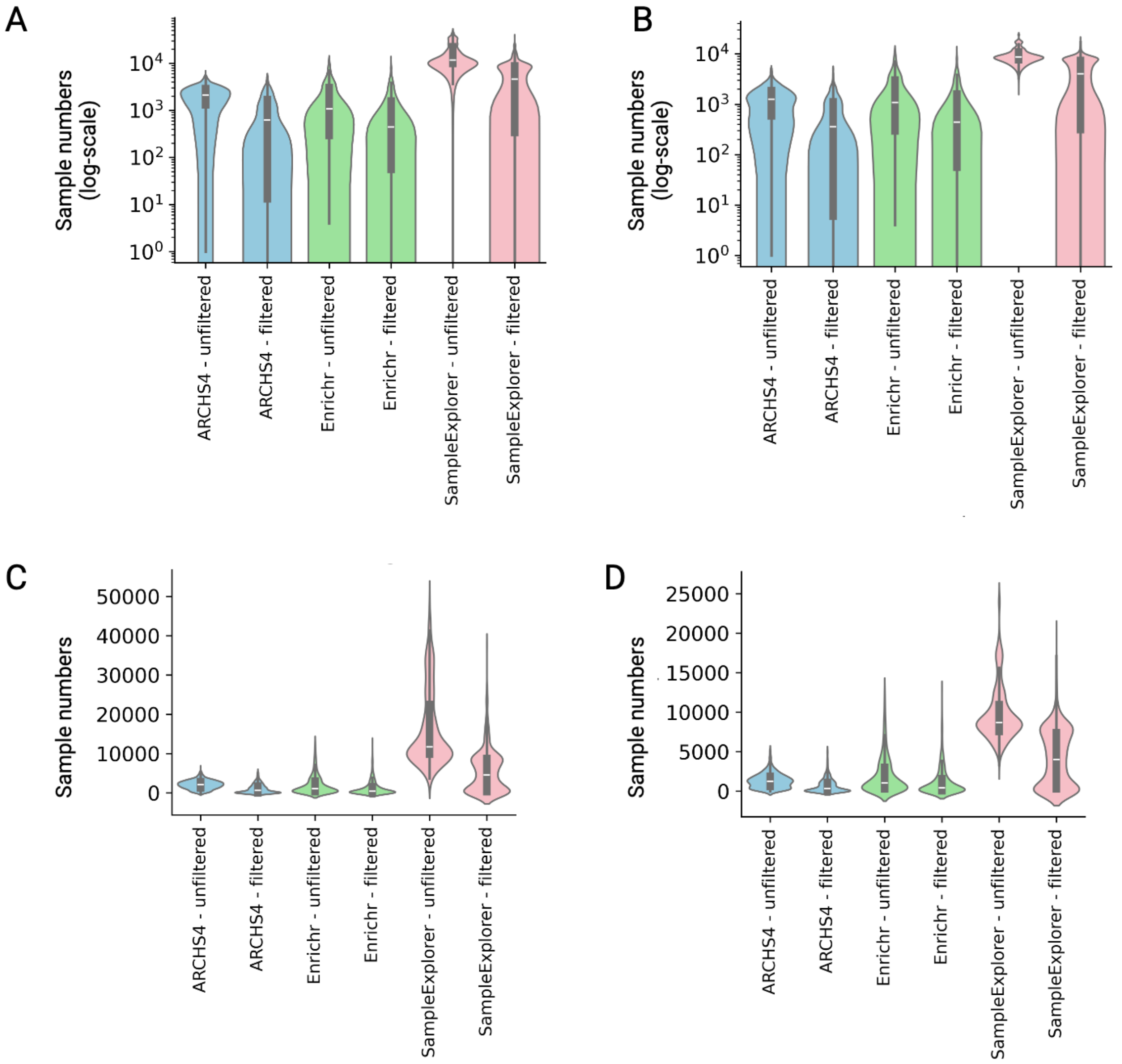

Supplementary Figure 5. Comparative analysis of sample retrieval across three platforms: ARCHS4, Enrichr, and SampleExplorer. Sample counts are shown on both logarithmic (A, B) and linear (C, D) scales. Upper panels (A, C) display total sample counts before excluding single-cell studies, while lower panels (B, D) show counts after filtering.

Tables and additional files

Dataset Name	Data Type	Description
channel_count	str	Number of channels per sample.
characteristics_ch1	str	Characteristics of channel 1.
contact_address	str	Address of the contact person/institution.
contact_city	str	City of the contact person/institution.
contact_country	str	Country of the contact person/institution.
contact_institute	str	Institute of the contact person.
contact_name	str	Name of the contact person.
contact_zip	str	ZIP code of the contact address.
data_processing	str	Details about data processing steps.
extract_protocol_ch1	str	Extraction protocol for channel 1.
geo_accession	str	GEO accession number.
instrument_model	str	Model of the instrument used.
last_update_date	str	Date of the last update.
library_selection	str	Library selection method.
library_source	str	Source of the library.
library_strategy	str	Strategy used for library preparation.
molecule_ch1	str	Type of molecule in channel 1.
organism_ch1	str	Organism information for channel 1.
platform_id	str	Platform identifier.
readsaligned	uint32	Number of reads aligned.
relation	str	Relation information.
sample	str	Sample identifier.
series_id	str	Series identifier.
singlecellprobability	float64	Probability related to single-cell data.
source_name_ch1	str	Source name for channel 1.
status	str	Status of the sample.
submission_date	str	Date of submission.
taxid_ch1	str	Taxonomy ID for channel 1.
title	str	Title or description of the sample.
type	str	Type/category of the sample.

Supplementary Table 1. Description of the datasets within the samples section of the HDF5 file. Each dataset contains attributes for 722,425 samples, with the data type specified. String data (str) represents textual information like sample characteristics and contact details, while numerical data (uint32 and float64) represent counts and probabilities. Each dataset captures key biological or technical aspects, such as organism, extraction protocols, or sequencing platform details.

Embedding	Context Precision	Context Relevancy
all-Mini-LM-v2	0.6950	0.2756
SciBERT	0.7300	0.2661
PubMedBERT	0.6919	0.2593
ada2-text-002	0.700	0.2621

Supplementary Table 2. Performance comparison of different embedding models for semantic search, measured by two key metrics: Context Precision and Context Relevancy. Context precision indicates the model's ability to retrieve semantically precise results in a relevant context, while Context relevancy reflects how

well the retrieved results align with the broader context of the query. The table compares all-Mini-LM-v2, SciBERT, PubMedBERT, and ada2-text-002 based on these metrics.

Strategies for comparison	Semantic search only	Semantic search with semantic expansion	Semantic search with transcriptome expansion	Enrichr API	ARCHS4 API
Enrichr API	0.9975	0.9950	0.9914	NA	0.9998
ARCHS4 API	0.9980	0.9965	0.9968	0.9998	NA

Supplementary Table 3. Normalised Jaccard distance (maximum = 1) comparison of samples retrieved using different semantic search strategies (with and without expansions), alongside API-based retrieval using the Enrichr and ARCHS4 APIs. Higher Jaccard distances indicate greater dissimilarity between sample sets. This table evaluates dissimilarity between search results produced by SampleExplorer using three different strategies and compares them with results retrieved from the Enrichr and ARCHS4 APIs. "NA" denotes that the specific comparison was not applicable or possible.

SampleExplorer strategies	Search step	Expand Step	Search step (N)	Expand Step (N)
S+S	Semantic	Semantic	50	5
S+T	Semantic	Transcriptome	50	10
T+T	Transcriptome	Transcriptome	1000	10
T+S	Transcriptome	Semantic	1000	5
S-only	Semantic	-	50	-
T-only	Transcriptome	-	1000	-

Supplementary Table 4. Parameters used in the SampleExplorer search strategies. The strategies combine semantic or transcriptome-based search and expansion steps, with "N" representing the number of samples retrieved at each step. The table outlines the search and expansion method (semantic or transcriptome) and the number of samples considered for each step. The labels correspond to those used in Figures 1C to 1F for comparative analysis.

Comparison	P-value	Interpretation
**Transcriptome-based retrieval (evaluation based on semantic similarity)**		
ARCHS4 vs SE (1-step)	6.70e-08	More closely related metadata in SE (1-step)
ARCHS4 vs SE (2-step)	2.44e-01	No significant difference
Enrichr vs SE (1-step)	9.16e-47	More closely related metadata in SE (1-step)
Enrichr vs SE (2-step)	2.69e-37	More closely related metadata in SE (2-step)
**Semantic retrieval (evaluation based on transcriptome-based similarity)**		
SE (1-step) vs ARCHS4	3.03e-13	Greater transcriptional diversity in SE (1-step)
SE (1-step) vs Enrichr	2.03e-02	Greater transcriptional diversity in SE (1-step)
SE (2-step) vs ARCHS4	1.89e-26	Greater transcriptional diversity in SE (2-step)
SE (2-step) vs Enrichr	3.56e-14	Greater transcriptional diversity in SE (2-step)

Supplementary Table 5: **Statistical comparison of average interpoint distances of returned samples across retrieval spaces and methods**. P-values from paired Wilcoxon tests comparing mean interpoint distances between methods. The tests evaluate whether the first method produces significantly larger average interpoint distances than the second method. SE - SampleExplorer.

Comparison	Transcriptome Space (ssGSEA metric) OR (p-value)	Semantic Space (Cosine Similarity metric) OR (p-value)	Interpretation
Enrichr vs SE (2-step)	0.525 (2.03e-04)	0.811 (2.26e-01)	Enrichr shows stronger correlation between low ssGSEA scores and tight interpoint distance; no significant relationship in semantic space
Enrichr vs SE (1-step)	0.279 (4.34e-18)	0.762 (1.49e-01)	Enrichr shows very strong correlation between low ssGSEA scores and tight interpoint distance; no significant relationship in semantic space

Comparison	Transcriptome Space (ssGSEA metric) OR (p-value)	Semantic Space (Cosine Similarity metric) OR (p-value)	Interpretation
ARCHS4 vs SE (2-step)	0.756 (8.29e-02)	0.949 (7.83e-01)	ARCHS4 shows weak correlation between low ssGSEA scores and tight interpoint distance; no significant relationship in semantic space
ARCHS4 vs SE (1-step)	0.777 (6.14e-02)	1.097 (6.42e-01)	ARCHS4 shows weak correlation between low ssGSEA scores and tight interpoint distance; no significant relationship in semantic space

Supplementary Table 6: **Associations between evaluation metrics and mean interpoint distances across retrieval methods**. Results of Fisher's Exact Tests show odds ratios (OR) and p-values for relationships between ssGSEA enrichment (transcriptome space) and cosine similarity (semantic space) with mean interpoint distances. OR < 1 indicates that lower metric scores are associated with smaller interpoint distances with respect to the first item in the comparison. SE - SampleExplorer; OR - Odds ratio.

Abbreviations

API	Application Programming Interface
GEO	Gene Expression Omnibus
HDF5	Hierarchical Data Format version 5
JL	Johnson-Lindenstrauss (transform)
LM	Language Model
ssGSEA	Single-sample Gene Set Enrichment Analysis

References

Benitez,J.A. *et al.* (2021) PTEN deficiency leads to proteasome addiction: a novel vulnerability in glioblastoma. *Neuro Oncol*, **23**, 1072–1086.

Clough,E. and Barrett,T. (2016) The Gene Expression Omnibus Database. *Methods Mol Biol*, **1418**, 93–110.

Glazko,G. and Mushegian,A. (2010) Measuring gene expression divergence: the distance to keep. *Biol Direct*, **5**, 51.
https://streamlit.io/.
Kuleshov,M.V. *et al.* (2016) Enrichr: a comprehensive gene set enrichment analysis web server 2016 update. *Nucleic Acids Res*, **44**, W90–W97.
Lachmann,A. *et al.* (2018) Massive mining of publicly available RNA-seq data from human and mouse. *Nat Commun*, **9**, 1366.
Li,P. *et al.* (2006) Very sparse random projections. In, *Proceedings of the 12th ACM SIGKDD international conference on Knowledge discovery and data mining*, KDD '06. Association for Computing Machinery, New York, NY, USA, pp. 287–296.
Liberzon,A. *et al.* (2011) Molecular signatures database (MSigDB) 3.0. *Bioinformatics*, **27**, 1739–1740.
Moravec,J. (2015) A Comparative Study: L1-Norm Vs. L2-Norm; Point-to-Point Vs. Point-to-Line Metric; Evolutionary Computation Vs. Gradient Search. *Applied Artificial Intelligence*, **29**, 164–210.
